# Biological evaluation of novel thiomaltol-based organometallic complexes as topoisomerase IIα inhibitors

**DOI:** 10.1007/s00775-020-01775-2

**Published:** 2020-03-19

**Authors:** Maria S. Legina, Juan J. Nogueira, Wolfgang Kandioller, Michael A. Jakupec, Leticia González, Bernhard K. Keppler

**Affiliations:** 1 Faculty of Chemistry, Institute of Inorganic Chemistry, University of Vienna, Vienna, Austria; 2 IADCHEM, Institute for Advanced Research in Chemistry, Universidad Autónoma de Madrid, Madrid, Spain; 3 Departamento de Química, Universidad Autónoma de Madrid, Madrid, Spain; 4 Research Cluster “Translational Cancer Therapy Research”, University of Vienna, Vienna, Austria; 5 Faculty of Chemistry, Institute of Theoretical Chemistry, University of Vienna, Vienna, Austria

**Keywords:** Topoisomerase IIα, Metal complex, Anticancer drug, Molecular dynamics, Monte Carlo simulation, RNA interference

## Abstract

**Abstract:**

Topoisomerase IIα (topo2α) is an essential nuclear enzyme involved in DNA replication, transcription, recombination, chromosome condensation, and highly expressed in many tumors. Thus, topo2α-targeting has become a very efficient and well-established anticancer strategy. Herein, we investigate the cytotoxic and DNA-damaging activity of thiomaltol-containing ruthenium-, osmium-, rhodium- and iridium-based organometallic complexes in human mammary carcinoma cell lines by means of several biological assays, including knockdown of topo2α expression levels by RNA interference. Results suggest that inhibition of topo2α is a key process in the cytotoxic mechanism for some of the compounds, whereas direct induction of DNA double-strand breaks or other DNA damage is mostly rather minor. In addition, molecular modeling studies performed for two of the compounds (with Ru(II) as the metal center) evinces that these complexes are able to access the DNA-binding pocket of the enzyme, where the hydrophilic environment favors the interaction with highly polar complexes. These findings substantiate the potential of these compounds for application as antitumor metallopharmaceuticals.

**Graphic abstract:**

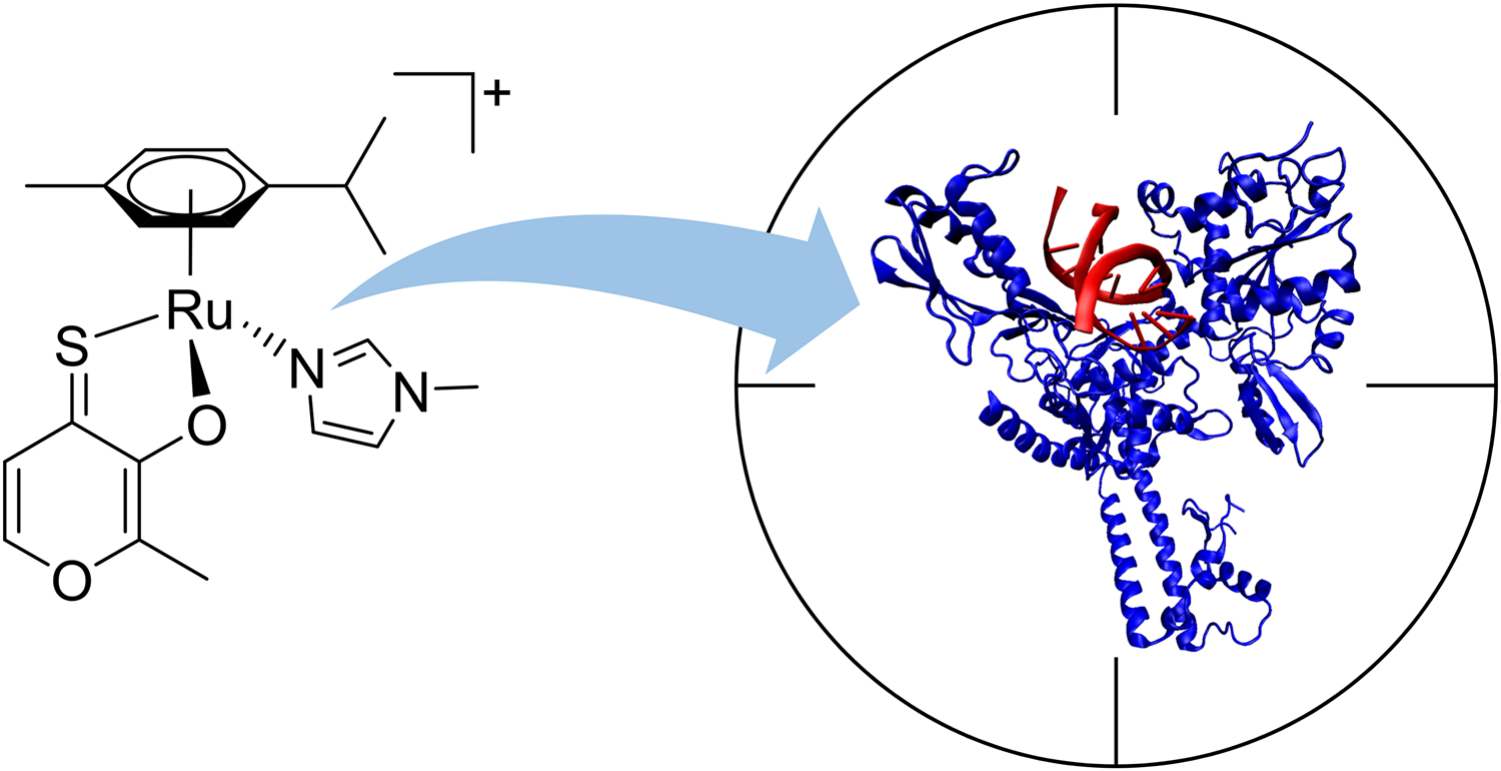

**Electronic supplementary material:**

The online version of this article (10.1007/s00775-020-01775-2) contains supplementary material, which is available to authorized users.

## Introduction

**Fig. 1:**
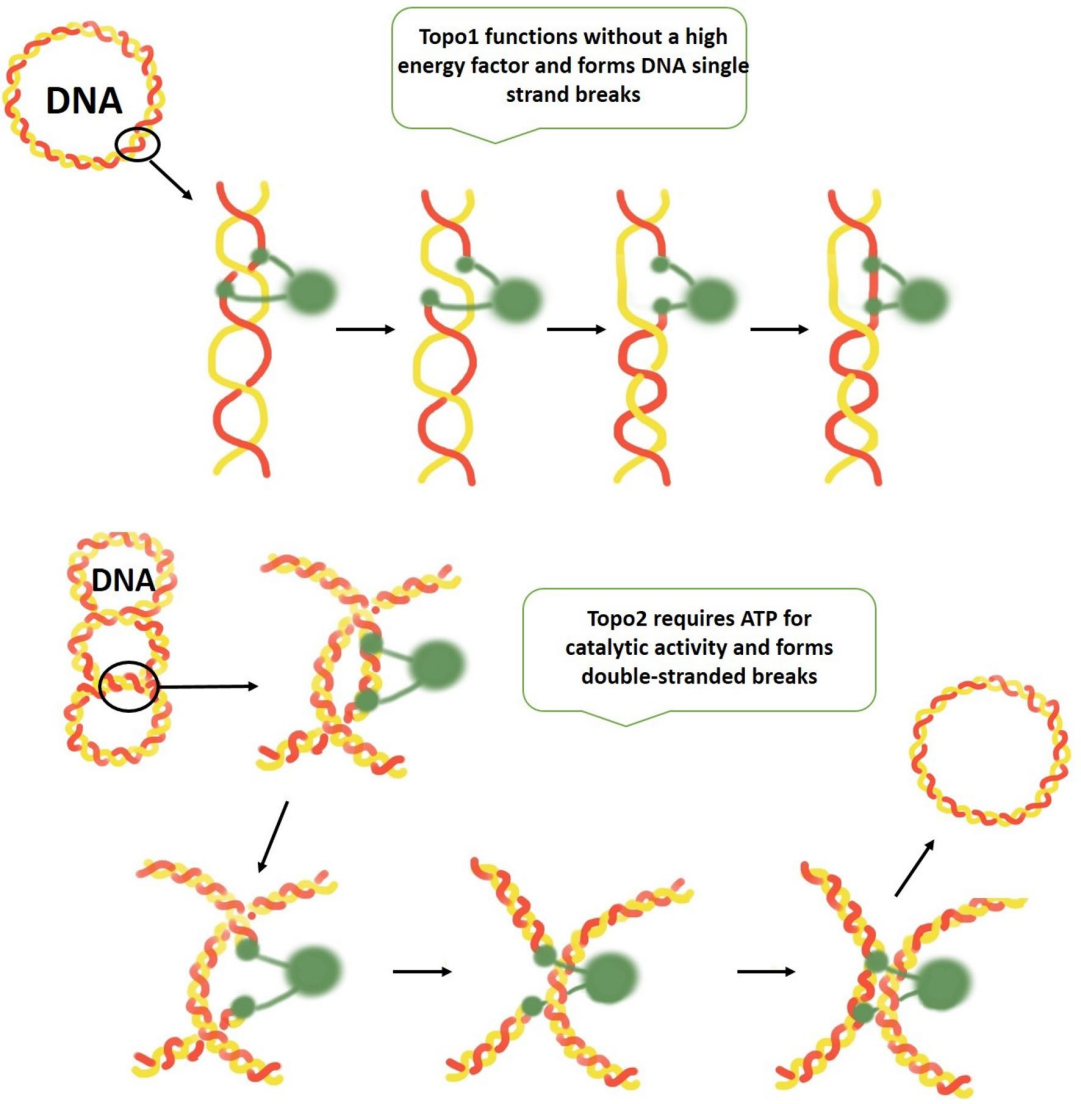
Catalytic activity of topo1 and topo2 enzymes—topo1 cuts one strand of the DNA double helix, whereas topo2 cuts both strands, requiring ATP (adenosine triphosphate), and is able to religate strands at the end of each cycle (designed based on a publication from John Nitiss) [14]

Since the discovery of the cytotoxic properties of cisplatin by Rosenberg in 1965 [1], the field of metal-based chemotherapeutic agents has been rapidly growing and developing every year [2]. Cisplatin, carboplatin, and oxaliplatin are the only FDA approved platinum drugs used worldwide for the treatment of various cancer types; however, there are many cases of resistance of tumors to platinum drugs. Drug resistance is a well-known phenomenon that results when diseases become tolerant to pharmaceutical treatments [3]. Some mechanisms of drug resistance are disease-specific, while others, such as the drug efflux observed in microbes and in human drug-resistant cancers, are evolutionarily conserved. Although many types of cancer are initially susceptible to chemotherapy, over time they can develop resistance through different mechanisms, such as DNA mutations and metabolic changes that promote drug inhibition and degradation [3]. The processes by which cells develop resistance to antitumor platinum drugs have been the subject of intense research [4] as it is a major obstacle for their clinical use. A large body of experimental evidence suggests that the antitumor activity of platinum complexes stems from their ability to form with DNA various types of covalent adducts [5, 6]; as a result, research on DNA modifications by these drugs and their cellular processing has predominated. The resistance of tumor cells to platinum drugs has been attributed to various processes, such as reduced platinum accumulation, intracellular inactivation, blocking of the induction of apoptosis, an increased repair of platinum–DNA adducts or a combination thereof [4, 5]. To overcome these obstacles, many different transition metal complexes have been synthesized and investigated [7]. In recent years, ruthenium-based molecules have emerged as promising antitumor agents. Certain ruthenium complexes possess unique biochemical features allowing them to accumulate preferentially in neoplastic tissues and/or to convert to their active state under specific pathophysiological conditions. In this respect, ruthenium (and osmium) compounds with their different mechanisms of action are promising candidates as anticancer drugs [8–10]. In the recent past, drug development has mainly relied on organic chemistry. This can be attributed to the lack of knowledge on the mechanisms and binding modes of metal-based drugs with biomolecular targets other than DNA. Traditionally, it was believed that the majority of metal-based drugs targeted DNA, but considerable evidence has accumulated that metal-based drugs are also able to bind to protein targets. Recent trends involve metal complexes that inhibit protein and lipid kinases, matrix metalloproteases, telomerases, topoisomerases, glutathione-S-transferases, and histone deacetylases [11–13]. In the present study, we investigate topoisomerases as targets for metal-based drugs. Topoisomerases are key nuclear enzymes that control the topological states of DNA by generating transient strand breakage. They are, therefore, involved in key processes such as DNA replication and transcription, as well as chromosome formation, enrichment, and separation (Fig. 1).

Mammalian cells encode two type II isozymes – topoisomerase IIα (topo2α) and β (topo2β), which have highly identical N-terminal ATPase and central core domains. However, they differ in their C-termini as well as in their expression patterns and cannot compensate for each other in vivo, i.e., if one of the forms is downregulated the other one cannot replace its functions equivalently. Moreover, the α isoform is produced primarily in the late S-phase, it remains associated with chromosomes at mitosis and plays a dominant role in this context, which makes it apparently more sensitive to pharmaceutical agents [15]. Well-known topo1-targeting drugs include camptothecin and its derivatives, while topo2-targeting drugs include doxorubicin, etoposide, and mitoxantrone. There are different types of topo2-targeting drugs, namely inhibitors and poisons. Topo2 poisons inhibit the enzyme’s activity by stabilization of the enzyme–DNA covalent complex, which leads to the accumulation of DNA double-strand breaks. In contrast, inhibitors of topo2 catalytic activity may stop the cycle by preventing binding of the enzyme to DNA, by blocking the ATP-binding site of the enzyme or by inhibiting the cleavage reaction. This kind of topo2-targeting drugs have become established in clinical practice – many studies have shown the efficacy of irinotecan (CPT-11, a derivative of camptothecin), which is approved by the FDA for use in colorectal cancer [14, 16–19]. The topo2α enzyme has been shown to be a proliferation marker associated with tumor grade and cell proliferation index [20]. The prognostic effect of topo2α seems different among different subtypes of breast cancer, but, in general, patients with high topo2α expression showed a significantly higher rate of distant metastasis and shorter distant metastasis free survival compared with patients with low topo2α expression [20]. Cancer cell lines expressing different levels of the enzyme would be a powerful tool in the investigation of topo2 behavior and responsiveness to treatment. The understanding of the molecular biology of cancer cell progression, invasion, metastasis, and failure to undergo cell death has led to a new generation of systemic anticancer therapies, which target specific cellular defects in malignant cells. Unlike classic cytotoxic chemotherapy, this new generation of drugs tends to work in tumors with specific genetic defects, allowing personalized treatment [21]. In this new era of therapies, topo2 targeting remains an indispensible anticancer strategy.

**Fig. 2:**
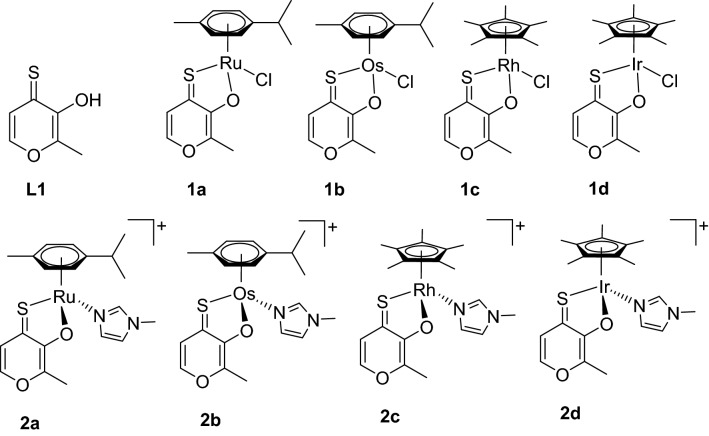
Structural formulas of tested compounds and ligand. Counterions (PF_6_^–^) of the charged complexes are omitted for clarity

Here we investigate the cytotoxicity of several thiomaltol-based metal complexes (Fig. 2) [22]. The chlorido complexes **1a–1d** hydrolyze quickly to the corresponding aqua species which is assumed to be the active form of these organometallics. As already reported, replacement of the labile chlorido ligand by *N*-methylimidazole remarkably increased the stability of **2a–2d**. However, the *N*-donor can be cleaved under acidic conditions, again yielding the more reactive aqua complex. All complexes exhibit remarkable cytotoxicity. Based on our findings in the aforementioned study, we assumed that inhibition of topoisomerase IIα might play a crucial role in the mode of action of organometallic thiomaltol complexes [22]. Within this work, we substantiate that topo2 is involved in the anticancer mode of action of some of these complexes and we unveil the interaction mechanism between the drugs and the enzyme by means of biological assays complemented with computational modeling. Given that topoisomerases are among the most well-established targets for anticancer therapy, this opens up new avenues for the development of this compound class.

## Results and discussion

Recently published findings [22] on the anticancer properties of novel Ru^II^, Os^II^, Rh^III^, and Ir^III^ thiomaltol complexes showed that they act as inhibitors of topo2 catalytic activity and have a significantly higher enzyme inhibitory capacity than the free ligand. In cell cycle studies the Ru^II^ and Ir^III^ methylimidazole-substituted complexes caused the highest S-phase accumulation (up to 43% and 45%, respectively), which was consistent with enzyme inhibition. Previously acquired data implied that the introduction of 1-methylimidazole as a leaving group substantially increased the stability in aqueous solution. It was suggested that this modification may allow accumulation of the intact complexes and controlled activation at the lower pH values within tumor tissues. The cytotoxicity of thiomaltol ligand **L1** and respective complexes **1a–d** and **2a–d** in A549, CH1/PA-1 and SW480 cells has already been reported [22]; however, new data for SK-BR-3, T47D, and MDA-MB468 (mammary carcinoma cells) were acquired within this work. In general, all complexes showed IC_50_ values in the low micromolar to high nanomolar range (Table 1). Remarkably, Os^II^ complexes are less active than the other complexes. The monodentate leaving group has only a marginal impact on the activity of the Os^II^ complexes, as the chlorido complex **1b** is only about 1.5 times more active than the 1-methylimidazole analog **2b** in most of the tested cell lines. In contrast, Rh^III^ complex **2c** was more active than its chlorido counterpart by factors of up to 2.6, depending on the cell line. The P-gp expressing cell line SW480 is mostly as sensitive to all the complexes as the broadly chemosensitive CH1/PA-1 cell line, whereas IC_50_ values in breast carcinoma cell lines (SK-BR-3, T47D and MDA-MB468) expressing different levels of topo2 enzyme are in a similar range with a few exceptions—Os^II^ complexes turned out to be up to tenfold less active and Ir^III^ complex **1d** is about twofold less active than in the former cell lines. In this respect, Ru^II^ analogs are in an intermediate position—they are more active than Os^II^ complexes but less active than the Ir^III^- or Rh^III^-based complexes with some deviations. In addition, the two Ru^II^ complexes differ from each other, with up to 4 times more activity for complex **2a** with respect to **1a**.

**Table 1:** Inhibition of cancer cell growth by tested substances in six human cancer cell lines; 50% inhibitory concentrations (means ± standard deviations), obtained by the MTT assay (exposure time: 96 h)

M	Compd	IC_50_ (µM, mean ± SD)					
		A549	CH1/PA-1	SW480	SK-BR-3	T47D	MDA-MB468
Ru^II^	**1a**	12 ± 4	3.3 ± 0.5	11 ± 1	16 ± 4	6.3 ± 1.7	7.2 ± 0.9
Os^II^	**1b**	4.1 ± 0.3	2.0 ± 0.2	2.0 ± 0.2	12 ± 3	13 ± 2	9.4 ± 0.9
Rh^III^	**1c**	5.9 ± 0.8	1.0 ± 0.1	1.0 ± 0.1	2.9 ± 0.8	1.8 ± 0.3	1.4 ± 0.2
Ir^III^	**1d**	5.8 ± 1.7	0.57 ± 0.03	0.73 ± 0.10	1.8 ± 0.6	1.3 ± 0.1	1.4 ± 0.1
Ru^II^	**2a**	7.1 ± 1.6	2.6 ± 0.5	3.0 ± 0.2	11 ± 4	4.4 ± 0.6	5.0 ± 0.4
Os^II^	**2b**	6.6 ± 0.4	3.0 ± 0.4	3.4 ± 0.7	30 ± 7	21 ± 4	37 ± 5
Rh^III^	**2c**	2.6 ± 0.6	0.86 ± 0.17	0.48 ± 0.03	1.1 ± 0.3	1.3 ± 0.1	0.53 ± 0.02
Ir^III^	**2d**	3.4 ± 0.8	0.63 ± 0.07	0.67 ± 0.04	1.6 ± 0.4	1.3 ± 0.6	0.70 ± 0.06
–	**L1**	1.3 ± 0.8	0.52 ± 0.04	0.55 ± 0.31	0.86 ± 0.05	0.46 ± 0.12	0.31 ± 0.04
–	Merbarone	n.d	9.2 ± 1.3	29 ± 3	22 ± 5	18 ± 2	17 ± 1
–	Etoposide	n.d	0.043 ± 0.010	0.20 ± 0.02	0.51 ± 0.06	0.59 ± 0.20	0.40 ± 0.08

Taking into account that we consider topo2 to be a target of these drugs, we had to make sure that there is no direct interaction with DNA. The ability to alter the secondary structure of DNA in cell-free experiments was studied by the use of an electrophoretic double-stranded DNA plasmid assay (Figure S1). In general, these metal-based complexes and the corresponding ligand **L1** did not show any impact on DNA mobility and its secondary structure; however, we saw an unusual behavior of plasmid DNA in the presence of some complexes—either the intensity of open circular (OC) band increased over the time, or additional bands between OC and supercoiled (SC) bands appeared. To make sure that this unusual pattern is not a result of the DNA breakage, we performed an H2AX assay that allows monitoring and accurately measuring phospho-specific histone H2AX activation in a population of cells. Histone H2AX functions downstream of the DNA damage kinase signaling cascade, and phosphorylation of this histone at serine 139 is an important indicator of DNA damage. As the level of DNA damage increases, the level of phospho-histone H2AX (also known as γH2AX) increases, accumulating at the sites of DNA damage. This accumulation is often used to indicate the level of DNA damage present within the cell [23, 24]. The evaluation of a formation of γ-H2AX in response to DNA double-strand breaks in SW480, SK-BR-3, T47D and MDA-MB468 cell lines after 48 h of exposure to the studied compounds showed no tremendous increase in DNA damage (Figure S2). Only Rh^III^ complexes **1c** and **2c** were clearly harmful for DNA and generated double-strand breaks in up to 33% of MDA-MB468 and up to 52% of SW480 cells (the latter cell line was the second most chemosensitive towards the tested substances over all cancer cell lines used). Ru^II^ complexes **1a** and **2a** generated a maximum of DNA damage in up to 13% of T47D cells. Probably due to the expression level of topo2α, T47D cells were relatively sensitive to all tested substances (9–30%) except ligand **L1** (DNA damage in 4% of cell populations). For comparison, DNA double-strand breaks were present in the negative control with 1–2% and in positive control (100 µM of etoposide) with 41–86%. It should be mentioned that in a previous publication the potential of these complexes to raise cellular ROS levels had been investigated. One of the well-known damaging effects of ROS is DNA damage, including double-stand breaks [25]. The obtained data indicated a slight increase in ROS levels for Rh^III^ complex **2c** by a factor 2.7, which may be a reason for the higher percentage of double-strand breaks in cells treated with this complex. To substantiate this finding, we investigated the ability of the Ru and Rh compounds to activate programmed cell death. Apoptosis/necrosis induction in SW480, SK-BR-3, T47D and MDA-MB468 cells after 48 h of exposure to the studied compounds and ligand, measured by flow cytometry using annexin V-FITC/propidium iodide double staining, demonstrated the ability of the complexes to induce cell death (Figure S3). Ru^II^ complexes **1a** and **2a** effectively induced apoptosis in SW480 and SK-BR-3 cells with 40–50 and 70–80%, respectively. Rh^III^ complexes **1c** and **2c** induced programmed cell death predominantly in SK-BR-3 cells with 70 and 88%, respectively. Surprisingly, ligand **L1** was able to induce apoptosis in SK-BR-3 and MDA-MB468 cells with 51 and 66%, respectively. For comparison, the positive control (160 µM of merbarone, as well-known inhibitor of topo2 catalytic activity) induced apoptosis only with up to 33%. The percentage of necrosis measured by this assay on average was no more than 12%. The fact that replacement of the chlorido ligand with methylimidazole (complex **2a**) increases both the stability of the complex in the presence of biomolecules and its solubility might explain why **2a** is more active than its analog **1a**. These two complexes also displayed different capacities of topo2α inhibition (as published previously)—**2a** complex was able to inhibit the enzyme activity at a concentration 4 times lower than the one required for its analog **1a** (2.5 vs 10 µM). Moreover, S-phase accumulation in the cell cycle studies (up to 45%) was consistent with enzyme inhibition experiments, and the capacity to induce apoptosis was 1.7-fold higher for **2a** than for **1a** [22]. Thus, we can conclude that rhodium complexes induce ROS activation that leads to DNA damage and apoptosis, while ruthenium complexes rather induce cell death via enzyme inhibition.

**Scheme 1:**
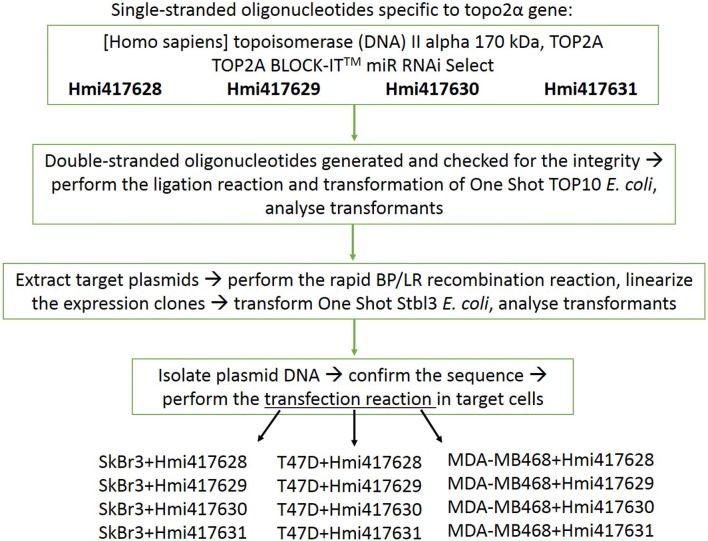
Generation of a cell line with knockdown of topo2α expression

Furthermore, we established methods to characterize anticancer properties of metal-based compounds with respect to the role of topo2α inhibition. One of the ideas that attracted our attention was that topo2α expression levels affect the cell response to drug treatment. Previous studies suggested that we may expect a different cell response to treatment in case of up/downregulation of topo2α expression levels. Some authors who have studied the effect of reducing the level of expression have shown that this can lead to either drug resistance or a higher susceptibility to drugs [25–28]. To investigate how different levels of topo2α expression may affect the cellular response to the treatment with the compounds studied here, we not only compared the cell lines with intrinsically different levels of topo2α expression but induced a knockdown of the enzyme expression by RNA interference (RNAi). RNAi is a biological process in which RNA molecules inhibit gene expression, typically by causing the destruction of specific mRNA molecules. This system is used to investigate the functions of different genes [29]. We used BLOCK-iT Expression Vector Kits that combine the advantages of traditional RNAi vectors—a stable expression and the ability to use viral delivery with capabilities for tissue-specific expression and multiple target knockdown from the same transcript. The pcDNA vectors of this kit are designed to express artificial miRNAs which are engineered to have 100% homology to the target sequence and will result in target cleavage. Since the complete inhibition of topo2α production in the cell leads to division failure and cell death [30, 31], we established several combinations of cell lines and miRNA sequences to reduce the production of the protein but not to inhibit it completely—the topo2α gene sequence was divided into four short sequences to produce RNA sequences complementary to different parts of the gene (see Scheme 1). Thus, we avoided complete inhibition of topo2α protein expression, as well as cell death. Still, topo2α knockdown variants of SK-BR-3 and MDA-MB468 cells stopped dividing or died on the following days. Therefore, only T47D-kn628 and T47D-kn630 cells were used for further experiments.

**Fig. 3:**
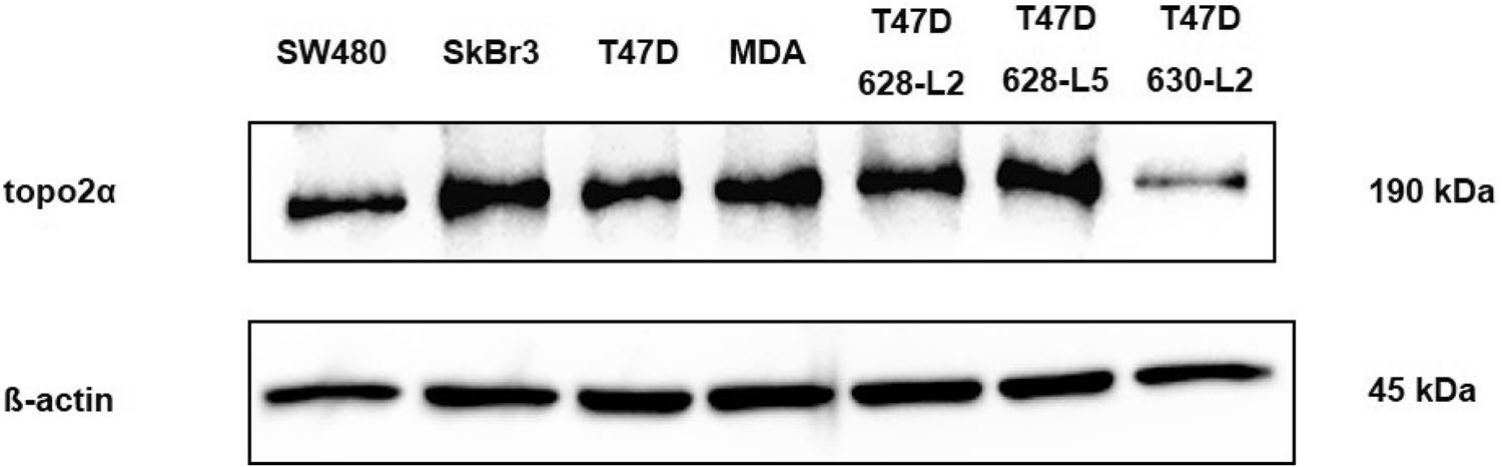
Western blotting visualization of topo2α protein level in SW480, SK-BR-3, T47D and MDA-MB468 standard cell lines as well as in the topo2α knockdown T47D cell lines (primers Hmi417628 and Hmi417630 – T47D-kn628 and T47D-kn630); β-actin was used as a loading control

The effect of RNAi was verified at the protein level by Western blotting and these experiments clearly showed the successful reduction of topo2α expression in one of the three variants – T47D-kn630 cells (Fig. 3). Then, the antiproliferative activity of the metal compounds was evaluated in the cell line T47D and its topo2α knockdown variant to investigate the possible role of the enzyme in the drug response (Table 2). The cell line with reduced level of topo2α expression T47D-kn630 turned out to be 2.3-fold more resistant to complex **2a** and 2.4-fold more resistant to ligand **L1**. These changes in response to treatment compared with the parental cell line T47D are more likely meaningful than the minor changes observed with other the compounds.

**Table 2:** Inhibition of cancer cell growth by tested substances in the human cancer cell line T47D and the knockdown variant of this cell line T47D-kn630; 50% inhibitory concentrations (means ± standard deviations), obtained by the MTT assay (exposure time: 96 h)

M	Compd	IC_50_ (µM, mean ± SD)	
		T47D	T47D-kn630
Ru^II^	**1a**	6.3 ± 1.7	3.8 ± 0.8
Ru^II^	**2a**	4.4 ± 0.6	10 ± 3
Rh^III^	**1c**	1.8 ± 0.3	1.9 ± 0.3
Rh^III^	**2c**	1.3 ± 0.1	0.80 ± 0.60
–	**L1**	0.46 ± 0.12	1.2 ± 0.5
–	Merbarone	18 ± 2	22 ± 3
–	Etoposide	0.59 ± 0.20	1.2 ± 0.3

Previously, the sensitivity to different anticancer drugs (amsacrine, doxorubicin, mitoxantrone, etoposide) was studied in a panel of breast cancer cell lines by Houlbrook and co-workers [15]. When the cells were ranked according to their sensitivity to one of the drugs, it was found that the ranking for other compounds did not follow the same pattern (with discrepancies from 2–6 times). Later experiments of Burgess and co-workers identified the topo2α expression level as the major determinant of response to the topoisomerase II poison doxorubicin and showed that suppression of the enzyme produces resistance to doxorubicin in vitro and in vivo [32]. They also mentioned the observation that the effects of topo2α knockdown were specific to topoisomerase II poisons, with shTop2A causing resistance to etoposide. A publication by Soubeyrand and co-workers reported that topoII siRNA ablation showed that etoposide cytotoxicity correlates with the inability of cells to correct topo2α-initiated DNA damage—these results linked the lethality of etoposide to the generation of persistent topo2α-dependent DNA defects within topologically open chromatin domains [33]. Logically, topo2α knockdown would be expected to confer resistance to a poison’s cytotoxicity, as decreased topoisomerase expression would produce fewer of the lethal DNA double-strand breaks that result from cleavage complexes. In case of inhibitors of topo2 catalytic activity (such as merbarone), it is not so easy to make any assumptions due to a limited number of published data. However, based on the IC_50_ values of Table 2, we can confirm that the sensitivity of the cells to the different drugs depends on the level of enzyme expression. Therefore, it is very likely that the topo2α enzyme is the target biomolecule of some of the compounds investigated here. However, the lack of a clear trend suggests that the interaction of metal complexes with topo2α is not the only cytotoxic mechanism and/or that the protein might play different roles in the mode of action of the drugs.

**Fig. 4:**
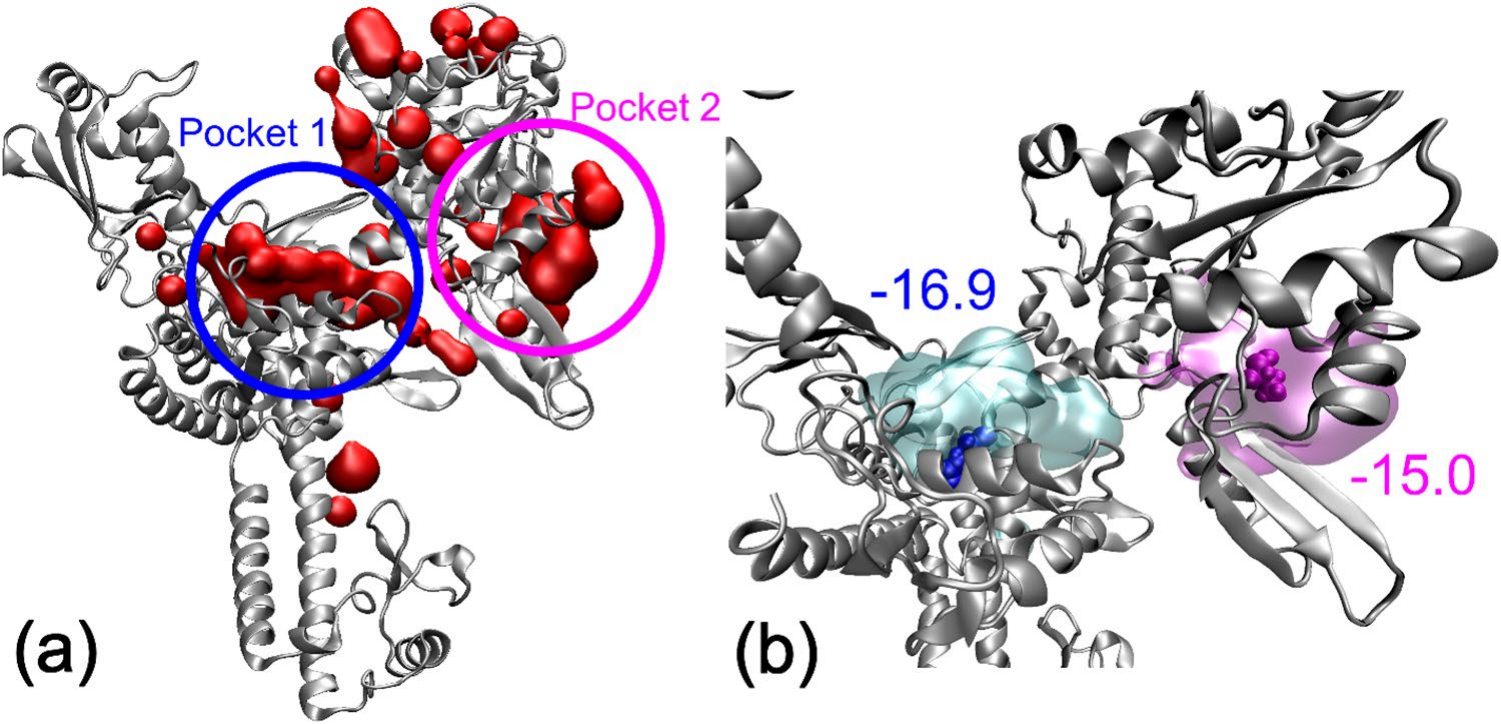
**a** Binding sites found along the classical MC simulation with free energies for the drug/protein binding process larger (in absolute value) than -15 kcal/mol represented by the red surface. Pockets 1 and 2 are schematically highlighted by blue and pink circles. **b** Binding pockets 1 and 2 represented by the cyan and magenta surfaces defined as the collection of binding sites located at a distance smaller than 15 Å from the residues PHE280 (blue van der Waals representation) and THR247 (magenta van der Waals representation), respectively. Binding free energies for each pocket in kcal/mol are also displayed. The protein is represented in silver color

Finally, we investigated the interaction of topo2α and the metal-based complexes by performing theoretical simulations. The first step of our simulations was to investigate the possible binding pockets of the DNA-binding domain of type II topoisomerase, where the thiomaltol ligand can be non-covalently bonded, by means of classical MC simulations. The calculation of the binding free energy along the MC simulation identified favorable binding sites. Figure 4a shows the binding sites of thiomaltol (represented by the red surface) on the surface of the protein for which the (absolute) binding free energy is larger than 15 kcal/mol. As can be seen, two main binding sites are revealed, namely pocket 1 and pocket 2, with the former being the DNA-binding site. A better characterization of both binding pockets can be obtained by computing the binding free energy for both pockets. For that purpose, the snapshots for which thiomaltol is in each of the binding pockets need to be identified; therefore, the region of the protein where the binding pockets are located must be (arbitrarily) defined. We have selected the residues PHE280 and THR247, which are shown in Fig. 4b by blue and magenta van der Waals spheres, respectively, as reference residues along the MC simulation. Then, if for a MC snapshot the center of mass of thiomaltol is separated from the center of mass of residue PHE280 (THR247) by less than 15 Å, it is considered that the ligand binds to pocket 1 (pocket 2). By employing this definition of the binding pockets, we have identified 199 and 205 binding events into pockets 1 and 2, respectively, with average binding free energies of − 16.9 and − 15.0 kcal/mol, as shown in Fig. 4b. This result indicates that the binding of thiomaltol to topoisomerase can occur in both pockets 1 and 2. However, the binding to pocket 1, which is the DNA-binding site, is slightly more thermodynamically favored.

In the next step, the two MC snapshots with the largest binding free energy for the pockets 1 and 2 were selected. For these two snapshots, the thiomaltol ligand is replaced by the Ru complexes **1a** and **2a** in both binding pockets. Thus, it is assumed that the binding pocket of the metal complexes is the same as that of the thiomaltol ligand. Then, classical MD simulations followed by QM/MM MD simulations are evolved for each of the four complex/pocket combinations. The binding affinity between the complexes and the protein could be analyzed by computing the intermolecular potential energy provided by the force field along the classical MD simulation. However, as discussed above, the force field employed here is not accurate enough for that purpose. Alternatively, the interaction energy between the complex and the protein could be calculated from the energy calculations along the QM/MM MD simulation. However, the present QM/MM computations do not allow the decomposition of the total interaction energy into Ru-complex/solvent and Ru-complex/protein contributions. Therefore, we analyze the binding affinity in terms of intermolecular contacts between the Ru species and the protein along the QM/MM MD simulation. The number of contacts, defined here as the number of interatomic distances smaller than 3 Å between the complexes and the protein, are listed in Table 3 for each binding pocket. As can be seen, in both binding pockets the total number of contacts for complex **2a** (61 for pocket 1 and 59 for pocket 2) is larger than that for complex **1a** (56 for pocket 1 and 51 for pocket 2). This suggests that intermolecular interactions between the Ru complexes and the protein are stronger for complex **2a** than for complex **1a**. In addition, the number of contacts per metal-complex atom, defined as the total number of contacts divided by the number of atoms of the metal complex, provides an estimation of the intermolecular interaction undergone by each atom of the complex.

**Table 3:** Average total number of contacts (water molecules) and average number of contacts (water molecules) per metal-complex atom between the Ru complexes and topoisomerase (in the solvation shell) within a cutoff distance of 3 Å computed along the QM/MM MD simulation

	Pocket 1		Pocket 2	
	Complex **1a**	Complex **2a**	Complex **1a**	Complex **2a**
Total contacts	56.3	61.5	51.2	58.6
Contacts per atom	1.3	1.2	1.2	1.1
Total waters	23.4	33.1	16.6	17.6
Waters per atom	0.55	0.65	0.40	0.35

As Table 3 shows, the number of contacts per atom is ca. 1.2 for both complexes and for both binding pockets, indicating that the strength of each Ru-complex/protein interatomic interaction is very similar in both complexes and pockets. By comparing the number of contacts for a particular complex in the different pockets, it is apparent that the intermolecular interactions are stronger for pocket 1 (56 contacts for complex **1a** and 62 for complex **2a**) than for pocket 2 (51 contacts for complex **1a** and 59 for complex **2a**). Therefore, based solely on the analysis of intermolecular contacts, the binding of complex **2a** into pocket 1 is the most favorable binding event.

**Fig. 5:**
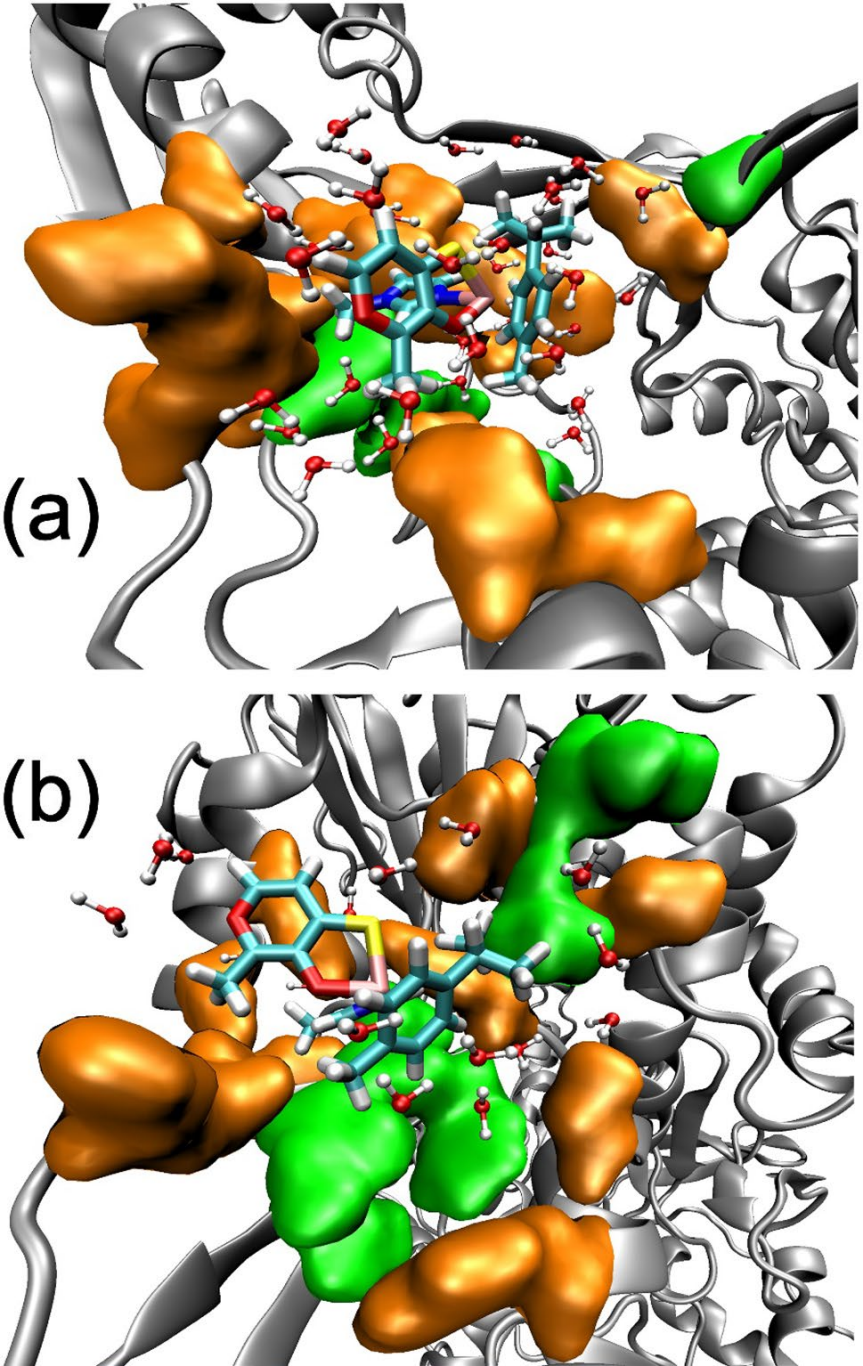
Polar and nonpolar amino acids represented in orange and green, respectively, that are located at a distance smaller than 6 Å from the metal complex **2a** in **a** pocket 1 and **b** pocket 2. The drug is displayed in tube representation with C in cyan, S in yellow, O in red, Ru in pink, and H in white. The water molecules solvating the complex located at a distance smaller than 3 Å from the complex are represented by balls and tubes, where oxygen is red and hydrogen is white. The protein is represented in silver color

The visualization of the QM/MM MD simulations reveals that the complexes embedded into the binding pockets are accompanied by a large solvation sphere, as can be seen in the representative snapshots displayed in Fig. 5 for the complex **2a** inside the pockets 1 and 2. The solvation sphere stabilizes the positive charge of the complexes and interacts with the polar amino acids of the protein, favoring the binding process. For both Ru compounds, the solvation sphere presents a larger number of water molecules in pocket 1 than in pocket 2. Specifically, considering a solvation shell of 3 Å from any solute atom, complex **1a** (**2a**) is solvated by 23 (33) and 17 (18) water molecules in pockets 1 and 2, respectively. Therefore, the desolvation process of the complexes, when going from the bulk solvent to the protein pocket, which contributes unfavorably to the binding process, is more important for pocket 2. The different degree of solvation in the two pockets can be understood by attending to the protein residues that surrounds the solvated complexes into the two binding sites. As Fig. 5a shows, pocket 1 presents a large number of polar amino acids (represented by the orange surfaces) at the binding site that create a polar environment that favorably interacts with the solvated complexes. Contrary, pocket 2 presents a more hydrophobic character at the binding site, allowing the penetration of fewer water molecules inside the pocket (Fig. 5b). This means that the energy penalty associated with the desolvation process is larger for pocket 2 than for pocket 1. Therefore, pocket 1 is the preferred binding site for the two Ru complexes. This means that complexes **1a** and **2a** could potentially prevent the binding of DNA to the DNA-binding site (pocket 1) of topo2α and inhibit the enzymatic activity of the protein.

Next, the different binding affinities between complexes **1a** and **2a** and the protein domain are examined by comparing the solvation spheres of both Ru compounds. In the preferred pocket 1, complex **2a** is solvated by a larger solvation sphere (33 water molecules) than complex **1a** (23 water molecules). This could seem an obvious conclusion since complex **2a** has a larger size than complex **1a** and, thus, a larger number of water molecules can interact with it. However, if the solvation spheres are compared in terms of number of water molecules per solute atom to eliminate the system-size dependency, the same conclusion is drawn. The solvation sphere of complex **2a** has 0.65 water molecules per solute atom, while that of complex **1a** has only 0.54 water molecules per solute atom when the compounds are embedded in pocket 1 (Table 3). The only structural difference between the two complexes is the substitution of water (complex **1a**) by 1-methylimidazole (complex **2a**), as shown in Fig. 2 (one should notice that the chlorido group of compounds **1a–d** is replaced by water after hydrolysis). The dipole moment of both ligands was computed by B3LYP/6-31G* using the optimized geometries at the same level of theory. 1-Methylimidazole presents a larger dipole moment (3.9 D) than water (1.9 D). Therefore, complex **2a** should be better solvated, in agreement with what is observed in the MD simulations. Moreover, the better solvated complex **2a** interacts stronger than complex **1a** with the DNA-binding site of topoisomerase, which presents a larger hydrophilicity than pocket 2. The hydrophobic environment found in pocket 2 induces a desolvation effect in both Ru species, which present solvation spheres of only 17 water molecules. Thus, the intermolecular contacts between the metal complexes and the protein and, most importantly, the analysis of the solvation spheres of the two complexes indicate that pocket 1 is the most likely binding site of the protein domain. In addition, complex **2a** interacts stronger with topoisomerase than complex **1a** and, thus, should be able to inhibit the DNA binding in a more effective way. This behavior is in consonance with the generally higher cytotoxicity experimentally observed for the complexes with the 1-methylimidazole ligand.

## Conclusion

The cytotoxic mechanism of several thiomaltol-based Ru, Os, Rh and Ir complexes has been investigated by means of biological assays and (for Ru complexes) theoretical simulations. Electrophoretic DNA plasmid and H2AX assays have demonstrated that some of the drugs do not induce a relevant degree of damage to the DNA structure and, thus, DNA is most likely not the main target of these drugs. On the other hand, the changes of IC_50_ values observed upon lowering topo2α levels by means of RNAi are consistent with the assumption that this enzyme is the main target at least for part of the compounds investigated here, primarily complex **2a** (in accordance with its higher topo2α inhibitory capacity shown previously [22]). Monte Carlo and molecular dynamics simulations have revealed that the metal compounds are able to bind into the DNA-binding pocket of the enzyme. In addition, the drugs enter the binding pocket with their solvation shell to maximize the interactions with the hydrophilic environment of the pocket. This indicates that the interactions between the metal complexes and the protein are not strong enough to compensate the energy penalty associated with the desolvation process of the drugs when they enter the binding pocket. Therefore, functionalization of the compounds with highly polar ligands favors their binding to the protein. This behavior is in agreement with the enhanced inhibition of cancer cell growth upon substitution of the labile chlorido ligand (which is readily exchanged for an aqua ligand in solution) by 1-methylimidazole. Our experiments also showed that inhibition of topo2α may not be the only way to inhibit cancer for this type of complexes. Thus, more research is required to validate the role of topo2α expression levels and investigate additional cytotoxic mechanisms. Nevertheless, these compounds may serve as potential pharmacophores for further rational design of inhibitors of topo2 catalytic activity. This adds a new aspect to the modes of action of experimental organometallics, which may allow overcoming some of the drawbacks of clinically applied metal-based anticancer agents.

## Experimental section

### Compounds

Thiomaltol complexes with ruthenium (Ru^II^), osmium (Os^II^), rhodium (Rh^III^), and iridium (Ir^III^), bearing either 1-methylimidazole or chloride as leaving group (Fig. 2), were synthesized and characterized according to procedures published elsewhere [22].

### Cell lines and culture conditions

SK-BR-3, T47D and MDA-MB468 (mammary carcinoma human cells) were kindly provided by Evelyn Dittrich (Department of Medicine I, Medical University of Vienna, Austria) and authenticated via STR profiling by Multiplexion, Heidelberg, Germany. All cell culture media and supplements were purchased from Sigma-Aldrich and plastic ware from Starlab. SK-BR-3 and SW480 cells were grown in 75 cm^2^ culture flasks in complete medium (i.e., Minimum Essential Medium supplemented with 10% heat-inactivated fetal bovine serum, 1 mM sodium pyruvate, 4 mM l-glutamine and 1% non-essential amino acids from 100 × stock), while T47D and MDA-MB468 cells in RPMI 1640 medium (supplemented with 10% heat-inactivated fetal bovine serum and 4 mM L-glutamine) as adherent monolayer cultures. After successful transfection the cell line T47D-kn was maintained in RPMI 1640 medium with 1.5% of antibiotic (blastidin) according to the manufacturer protocol (Invitrogen by life technologies). Cultures were grown at 37 °C under a humidified atmosphere containing 5% CO_2_ and 95% air.

### Antiproliferative activity assay (MTT)

Antiproliferative activity in vitro was determined by the MTT assay (MTT = 3-(4,5-dimethyl-2-thiazolyl)-2,5-diphenyl-2H-tetrazolium bromide). For this purpose, cells were harvested from culture flasks by use of trypsin and seeded in appropriative medium (100 µL/well) into 96-well plates in densities of 5 × 10^3^ (SK-BR-3), 8 × 10^3^ (T47D) and 2 × 10^3^ (MDA-MB468) as well as 24 × 10^3^ (T47D-kn) cells per well (growth kinetic tests were performed to estimate and confirm the required number of cells for further experiments). Cells were allowed for 24 h to settle and resume proliferation. Test compounds were dissolved in DMSO first, diluted in appropriate medium and instantly added to the plates (100 µL/well), where the DMSO content did not exceed 0.5%. After exposure for 96 h, the medium was removed and replaced with 100 μL/well of a 1:7 MTT/RPMI 1640 solution (MTT solution, 5 mg/mL of MTT reagent in phosphate-buffered saline; RPMI 1640 medium) and incubated for 4 h at 37 °C. Subsequently, the MTT/RPMI 1640 solution was removed, and the formazan product formed by viable cells was dissolved in DMSO (150 µL/well). Optical densities were measured with a microplate reader (BioTek ELx808) at 550 nm (and a reference wavelength of 690 nm) to yield relative quantities of viable cells as percentages of untreated controls, and 50% inhibitory concentrations (IC_50_) were calculated by interpolation. Evaluation is based on at least three independent experiments with triplicates for each concentration level.

### Flow cytometric detection of apoptotic/necrotic cells

Induction of cell death was analyzed by flow cytometry using FITC-conjugated annexin V (BioVision, USA) and propidium iodide (PI, Fluka) double staining. SK-BR-3, T47D, MDA-MB468 and SW480 cells were seeded into 12-well plates in a density of 5–13 × 10^4^ cells per well in complete medium and allowed to settle for 24 h. The cells were exposed to test compounds in different concentrations for 48 h at 37 °C. Merbarone (Sigma-Aldrich) was used as a positive control at a concentration of 160 µM. After incubation, cells were gently trypsinized, washed with PBS, and suspended with FITC-conjugated annexin V (0.25 μg/mL) and PI (1 μg/mL) in binding buffer (10 mM HEPES/NaOH pH 7.4, 140 mM NaCl, 2.5 mM CaCl_2_) at 37 °C for 15 min. Stained cells were analyzed with a Guava 8HT EasyCyte flow cytometer (Millipore) using InCyte software and analyzed by FlowJo software. Results are based on three independent experiments.

### Plasmid DNA interaction studies

pUC19 DNA (2686 bp) plasmid was purchased from Fermentas Life Sciences. 400 ng of pUC19 plasmid were incubated with 10 μM of the test compounds in 0.1 × Tris–EDTA (TE) buffer for different time intervals (30 min up to 8 h) at 37 °C. Electrophoresis was performed in agarose (from Sigma-Aldrich) gel 1% w/v in 1 × Tris–borate-EDTA (TBE) buffer for 90 min at 80 V. Gels were stained with ethidium bromide (EtBr) in 1 × TBE (0.75 μg/ml) for 20 min. Images were taken with the multi-imaging detection system Fusion SL (Vilber Lourmat). Results are based on two independent experiments.

### DNA damage detection assay (γH2AX)

Evaluation of a formation of γ-H2AX in response to DNA double stranded breaks was performed with the Flow Cellect™ Histone H2A.X Phosphorylation Assay Kit (Millipore). SK-BR-3, T47D, MDA-MB468 and SW480 cells were seeded into 6-well plates in a density of 15–25 × 10^4^ cells per well in complete medium and allowed to settle for 24 h. The cells were exposed to test compounds at different concentrations for 48 h at 37 °C. Etoposide (Sigma-Aldrich) was used as a positive control at a concentration of 100 µM. All following steps for DNA damage evaluation were performed according to the manufacturer’s protocol. Numbers of cells and their viability state were determined with a Guava 8HT EasyCyte flow cytometer (Millipore) using ViaCount software. Results are based on three independent experiments.

### Knockdown of topo2α expression level by RNAi

Single-stranded oligonucleotides specific for the topo2α gene were purchased from Invitrogen and the knockdown of topo2α expression level by RNAi was performed according to manufacturer’s protocol. Details of the single-stranded oligonucleotides specific for the topo2α gene: [Homo sapiens] topoisomerase (DNA) II alpha 170 kDa, TOP2A – TOP2A BLOCK-IT™ miR RNAi Select Hmi417628; Hmi417629; Hmi417630; Hmi417631. Double-stranded oligonucleotides were generated and checked for integrity, then ligation reaction and transformation of One Shot TOP10 *E. coli* was performed and transformants analyzed. Target plasmids were extracted, rapid BP/LR recombination reactions performed, expression clones linearized, One Shot Stbl3 *E. coli* transformed and transformants analyzed. Plasmid DNA was isolated, the sequence confirmed and transfection reaction in target cells SK-BR-3, T47D and MDA-MB468 performed. The gene knockdown was performed as described in the manufacturer’s protocols and illustrated in Scheme 1.

In the next few days topo2α knockdown versions of SK-BR-3 and MDA-MB468 cells stopped division or died. T47D cells with sequences Hmi417628 (T47D-kn628) and Hmi417630 (T47D-kn630) were checked for the verification of topo2α knockdown via Western blotting.

### Western blotting

The visualization of topo2α protein level in SW480, SK-BR-3, T47D and MDA-MB468 cells as well as the verification of topo2α knockdown in T47D-kn628/630 cells was performed by Western blotting. Cells were seeded in densities of 1.5–2 × 10^5^ cells per well into 6-well plates (Starlab) and allowed to resume proliferation for 24 h. After the cells were washed with PBS and lysed by adding 100 µL per well of RIPA lysis buffer (150 mM sodium chloride, 1.0% Triton X-100, 0.5% sodium deoxycholate, 0.1% SDS (sodium dodecyl sulfate), 50 mM Tris, pH 8.0). Cells were carefully scratched and sonicated for 10 s to shear DNA and reduce sample viscosity. The protein content of lysates was measured with the Pierce Micro BCA Protein Assay (Thermo Scientific). The appropriate volume of cell lysates (20 µg protein content per gel pocket) was mixed with 6ʹ loading buffer (12% w/v SDS, 30% 2-mercaptoethanol, 60% glycerol, 0.012% bromophenol blue, 0.375 M Tris HCL, pH 6.8) and heated to 95 °C for 5 min. The proteins were separated by 8% SDS–polyacrylamide gel electrophoresis and subsequently transferred onto nitrocellulose membranes (Millipore) using a semi-dry blotting apparatus (Biorad). The membranes were blocked with blocking buffer (1´ TBS, 0.1% Tween-20 with 5% BSA) and immunoblotted with the relevant primary topo2α rabbit antibody diluted in TBS/T buffer in a ratio of 1:1000 (Cell Signaling Technology). Primary antibodies were detected using anti-rabbit IgG HRP-linked antibody diluted in TBS/T buffer in a ratio of 1:3000 (Cell Signaling Technology) and visualized with the chemiluminescence detection system Fusion SL (Vilber Lourmat) using SuperSignal West Pico Chemiluminescent Substrate (Thermo Scientific). Afterwards, the membranes were cleaned by stripping buffer (15 g glycine, 1 g SDS, 10 mL Tween 20, pH 2.2) and immunoblotted with the β-actin rabbit antibody diluted in TBS/T buffer in a ratio of 1:3000 (Cell Signaling Technology) to check the quality of gel loading. Results are based on two independent experiments.

### Computational details of binding of thiomaltol complexes to topo2α

Since all metal complexes investigated here are positively charged (the chlorido ones after hydrolysis), they may potentially bind to topo2α into a pocket with large polarity, as it is the case of the DNA-binding domain of the protein. To explore this possibility, the binding affinity between the complexes **1a** and **2a** (see Fig. 2) and the DNA-binding domain of topo2α (PDB code 3L4K) [22] has been theoretically investigated by means of Monte Carlo (MC) and Molecular Dynamics (MD) simulations following the protocol explained below.

First, the DNA helix that is present in the crystal structure was manually removed. Then, the protein was protonated and solvated by a periodic truncated octahedral box of water molecules extended to a distance of 12 Å from any solute atom by the leap module of AmberTools15 [35]. This results in a solvated protein with one positive charge that was neutralized with one chloride ion. The solvated protein was minimized for 20,000 steps, where the first 10,000 steps were driven by a steepest-descent algorithm, and the last 10,000 steps by a conjugated-gradient algorithm. The system was then heated in the canonical ensemble (NVT ensemble) from 0 to 300 K by a classical MD simulation for 1 ns using a time step of 2 fs. During the heating process the motion of the protein was restrained with a harmonic force constant of 10.0 kcal/(molÅ^2^). Then, a classical MD simulation was run in the NVT ensemble at 300 K for 1 ns without applying any restrains to the motion of the protein. After heating, the density of the solvent and the structure of the protein was equilibrated in the isothermal–isobaric ensemble (NPT) during a 50 ns simulation with a time step of 2 fs. The Berendsen barostat with a pressure relaxation time of 2 ps was used to maintain a pressure of 1 bar. The Langevin thermostat with a collision frequency gamma of 1.0 ps^−1^ was used in both heating and equilibration steps to control the temperature. Moreover, the bond distances involving H atoms were restrained by the SHAKE algorithm [36]. Along the whole protocol the Coulomb and van der Waals interactions were truncated at 10 Å. The Coulomb interactions were evaluated by the particle mesh Ewald method [37] using a grid spacing of 1 Å in each direction for the charge grid, in which the reciprocal sums are computed by a fourth-order interpolation, and a direct sum tolerance of 10^–5^. The whole system, i.e., the protein, water molecules, and chloride ion were described by a force field [38–40]. All these steps were run by the Amber14 package [35].

The last snapshot from the previous classical MD simulations, for which the solvated protein is well equilibrated, was selected and the water molecules and the chloride ion were removed. Then, the different binding pockets of the equilibrated protein have been explored by a classical Monte Carlo (MC) simulation of 4000 steps using the Protein Energy Landscape Exploration (PELE) algorithm [41]. Since accurate force fields for Ru-based complexes are not available in the literature, the binding pockets of the protein have been explored by the thiomaltol ligand, which is present in all the metal complexes investigated here. Therefore, it was assumed that the binding mode of the metal complexes is the same as that of thiomaltol. Due to its small size and rigidity, the thiomaltol ligand was treated as a rigid body. The OPLS-AA force field [42] was used to describe topoisomerase and thiomaltol, while the aqueous solvent was modeled by the implicit surface-general Born continuum model [43]. The default values for the MC parameters implemented in the PELE algorithm [41] were used. The calculation of the binding free energy between thiomaltol and the protein for each of the 4000 MC steps allowed the identification of two favorable binding pockets (see below). These pockets will be named here pocket 1 and pocket 2, with the first one being the DNA-binding pocket.

In the next step, the thiomaltol ligand bound to the two favorable binding pockets has been replaced by the hydrolyzed Ru complex **1a** and by the Ru complex **2a** such that the thiomaltol ligands of the complexes are aligned with the isolated thiomaltol ligand inside the pockets to keep the same binding pose. This results in the generation of four different systems: complex-**1a**/pocket-1, complex-**1a**/pocket-2, complex-**2a**/pocket-1, and complex-**2a**/pocket-2. The metal-complex/topoisomerase systems were neutralized by adding two chloride ions and solvated by a periodic truncated octahedral box of water molecules extended to a distance of 12 Å from any solute atom by the leap module of AmberTools15 [35]. Then, the system was classically minimized for 5000 steepest-descent and 5000 subsequent conjugated-gradient steps. After minimization, a classical MD simulation in the NVT ensemble was run for 20 ps with a time step of 1 fs to heat the system up to 300 K. Afterwards, a simulation in the NPT ensemble was run for 1 ns to equilibrate the density of the solvent and to accommodate the metal complexes inside the binding pockets of the protein. As mentioned above, accurate force fields are not available in the literature for Ru complexes, especially when metal/π interactions are important, as it is the case here. Therefore, the internal coordinates of the complex were frozen during the classical simulations. This means that intramolecular parameters describing the internal motion of the Ru species are not necessary. However, point charges and Lennard–Jones parameters are still needed to describe the non-bonded interactions between the Ru complexes and the solvated protein environment. The Lennard–Jones parameters for the Ru atom were taken from an MM3 force field developed for Ru(II)-polypyridyl complexes [44], while the Lennard–Jones parameters for the remaining metal-complex atoms were taken from the General Amber Force Field [45]. The charges were derived by, first, optimizing the geometries of the complexes **1a** and **2a** quantum mechanically by density-functional theory (DFT) using the Gaussian09 program [46]. Specifically, the B3LYP functional [47–50] with the D3BJ dispersion correction [51] and the LanL2DZ effective-core potential [52] for Ru and the 6-31G* basis set [53] for the remaining atoms were employed. Then, the Mulliken charges were computed for the optimized geometries at the same level of theory.

In the last step of our protocol, the last snapshot of each of the four previous classical MD simulations (one for each complex/pocket combination) were taken as initial conditions for quantum mechanics/molecular mechanics (QM/MM) MD simulations in the NPT ensemble for 10 ps with a time step of 2 fs. The same thermostat and barostat used in the classical simulations were used here. The metal complexes **1a** and **2a** were described quantum mechanically by the same density functional, basis set, and effective-core potential specified above using TeraChem1.9 [54, 55] through the interface to external QM programs implemented in Amber14 [35] and run at GPUs. Dispersion corrections were considered by the D3 parameterizations of Grimme and coworkers [56]. The protein, water molecules, and chloride ions were treated by the force field mentioned above. The interaction between the QM and MM region layers was computed by an electrostatic embedding [57]. The Coulomb and van der Waals interactions were truncated at 8 Å and a real-space cutoff was used to compute the long-range QM–QM and QM–MM electrostatic interactions. These simulations allow the relaxation of the structure of the Ru complexes at QM level and, thus, provide a better description of the vibrational motion of the complexes and their interaction with the binding pockets of topoisomerase and with the solvent. From the QM/MM MD simulations 2000 snapshots were printed for analysis. The number of contacts between the Ru complexes and the protein and the number of water molecules in the solvation shells of the Ru complexes (see below) were obtained by the CPPTRAJ module [58] implemented in AmberTools15 [35]. The visualization of the MC and MD simulations and the graphical representations of the systems were carried out by the Visual Molecular Dynamics (VMD) program [59].

## Electronic supplementary material

Below is the link to the electronic supplementary material.

Supplementary file1 (DOCX 944 kb)
