# Supplementary material for: Biological evaluation of novel thiomaltol-based organometallic complexes as topoisomerase IIα inhibitors
Source: J Biol Inorg Chem. 2020 Mar 19;25(3):451–65. doi: 10.1007/s00775-020-01775-2 (PMC7186247; doi:10.1007/s00775-020-01775-2)
Supplement: Supplementary file 1 — Supplementary file1 (DOCX 944 kb) [file 775_2020_1775_MOESM1_ESM.docx]

SUPPORTING INFORMATION

Biological evaluation of novel thiomaltol-based organometallic complexes as topoisomerase IIα inhibitors

Maria S. Legina, Juan J. Nogueira, Wolfgang Kandioller, Michael A. Jakupec, Leticia González and Bernhard K. Keppler

Table of content

Plasmid DNA interaction studies S2

DNA damage detection assay (γH2AX) S3

Flow cytometric detection of apoptotic/necrotic cells S7

**
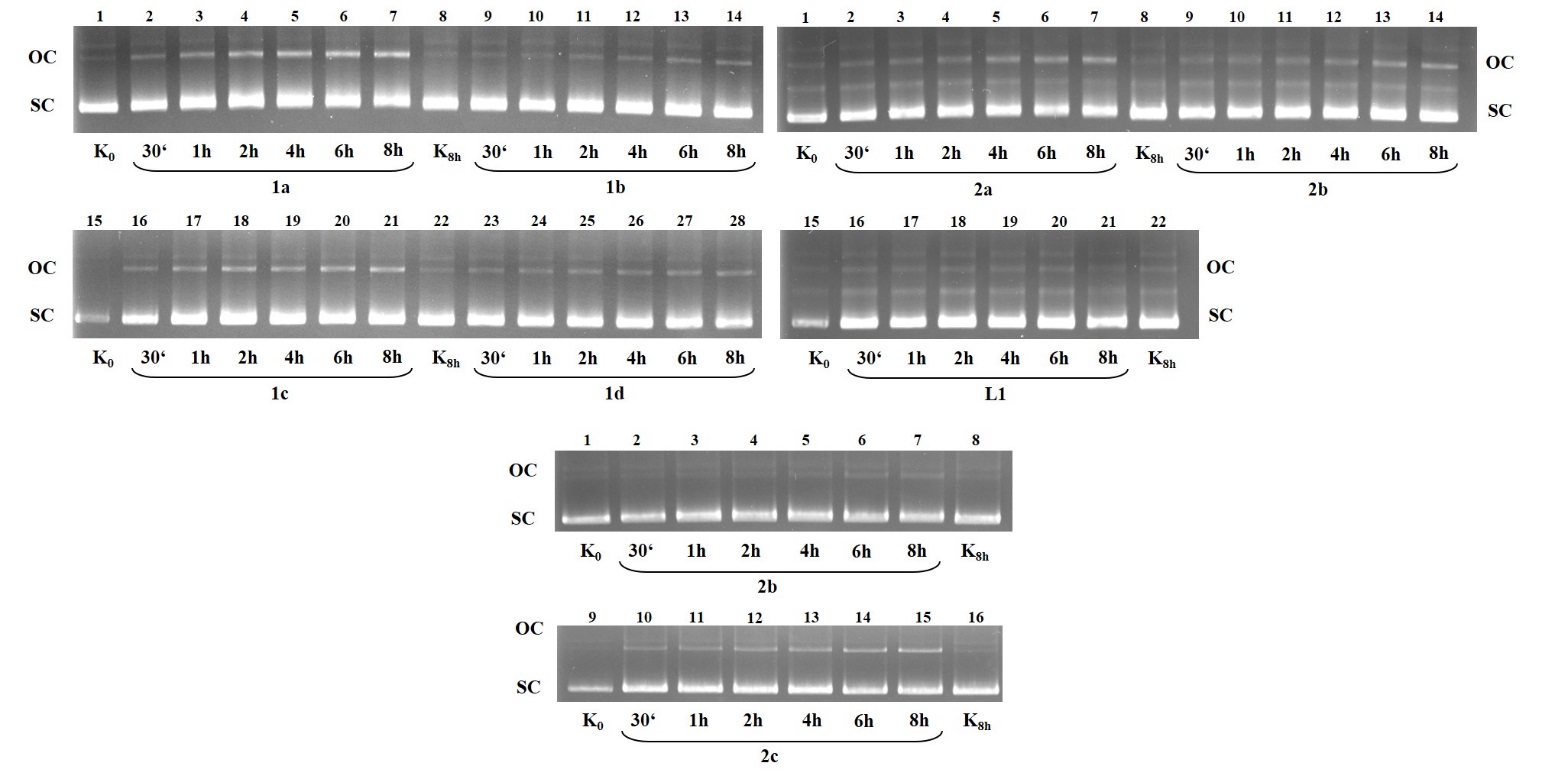
Figure S1.** Electropherograms of dsDNA plasmid pUC19 after exposure to 10 μM solutions of **1a**, **1b**, **1c**, **1d**, **2a**, **2b**, **2c**, **2d** and **L1** for different exposure times (30 min to 8 h) compared to untreated controls K_0_ and K_8h_.


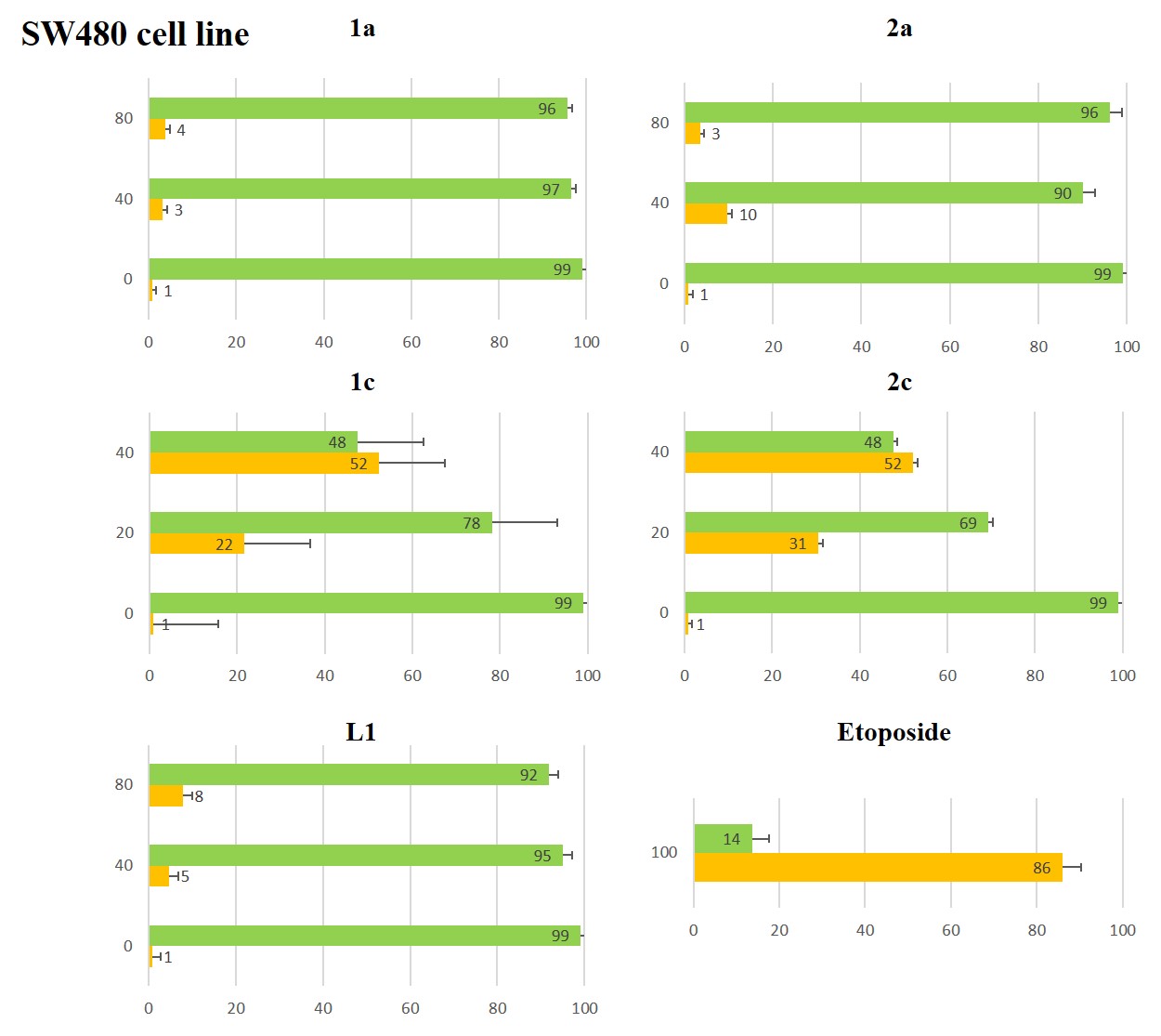


Figure S2. Evaluation of a formation of γ-H2AX in response to DNA double-strand breaks (DSBs) in SW480, SK-BR-3, T47D and MDA-MB468 cells after 48 h of exposure to the studied compounds and ligand; measured by flow cytometry using the γ-H2AX assay (undamaged cells shown in green, damaged – in yellow).


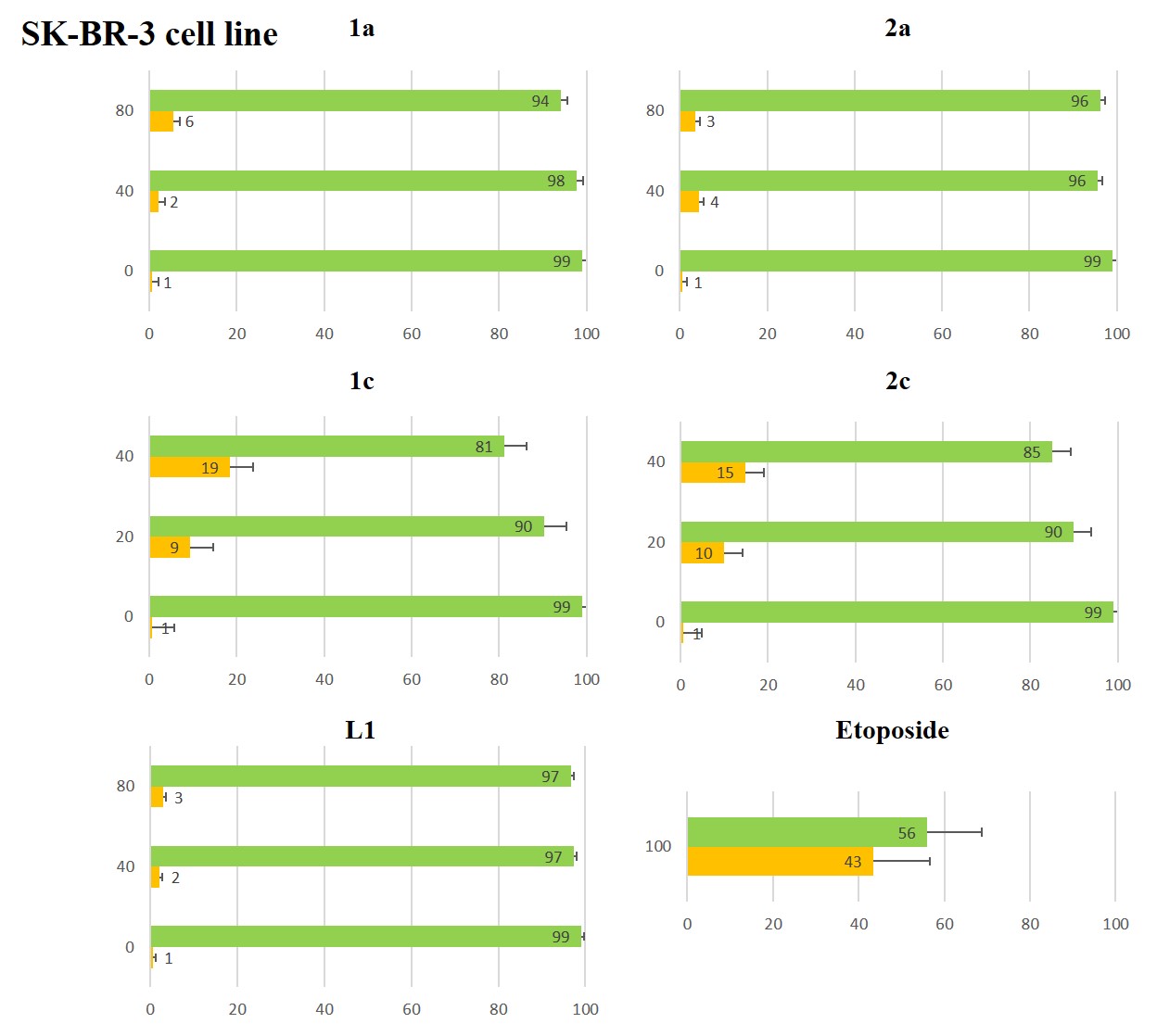


**Figure S2** (continued)


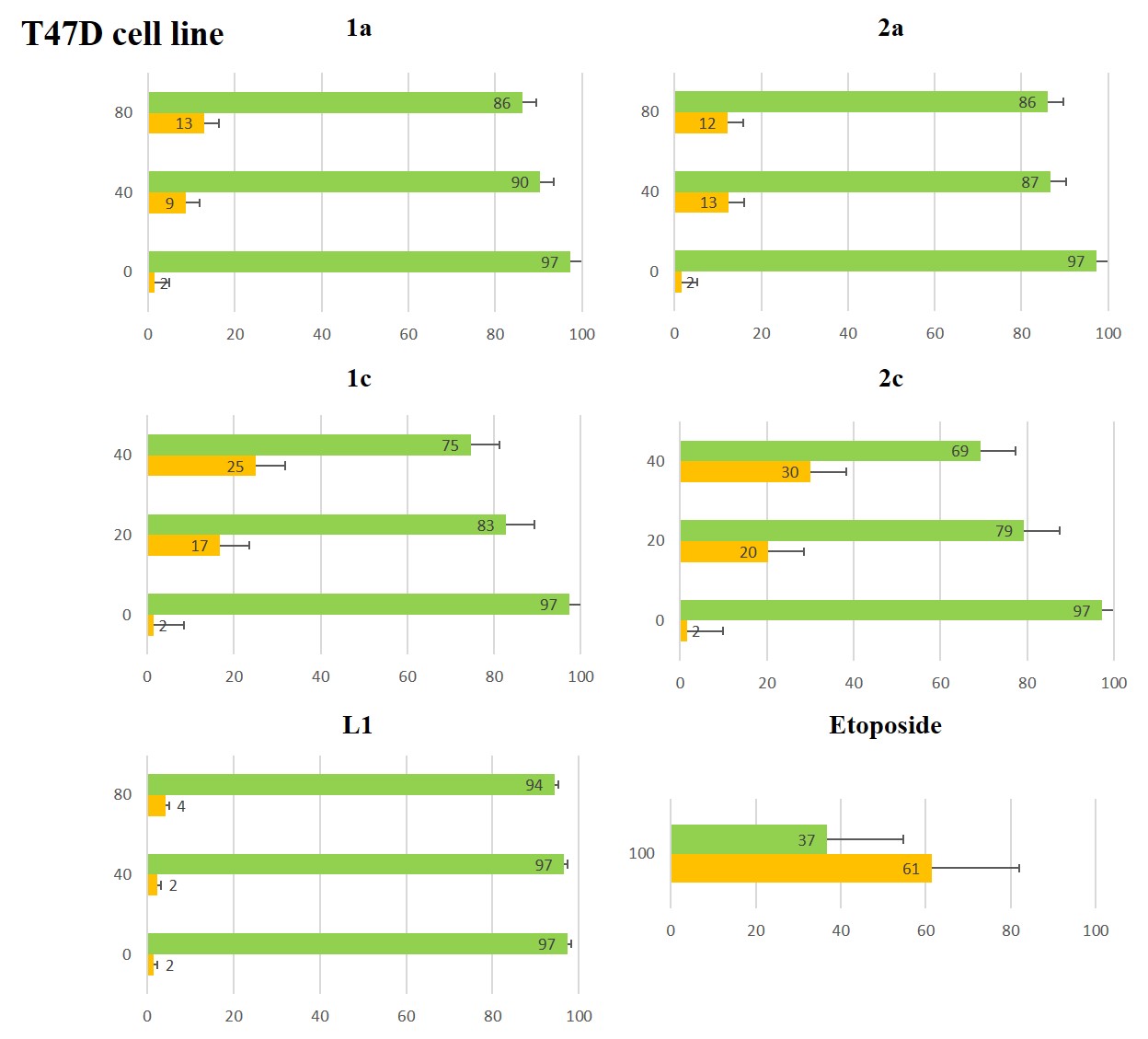


**Figure S2** (continued)


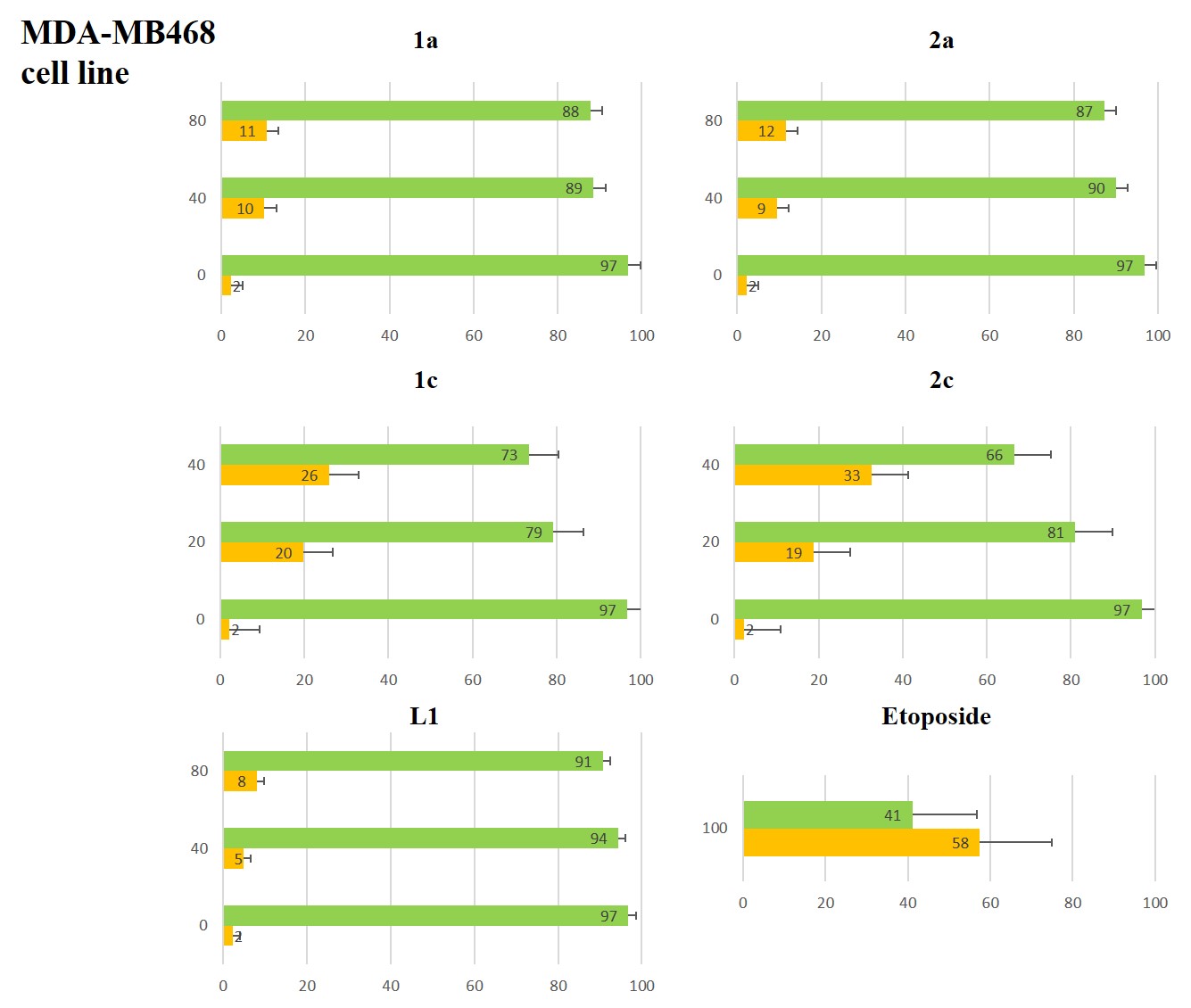


**Figure S2** (continued)


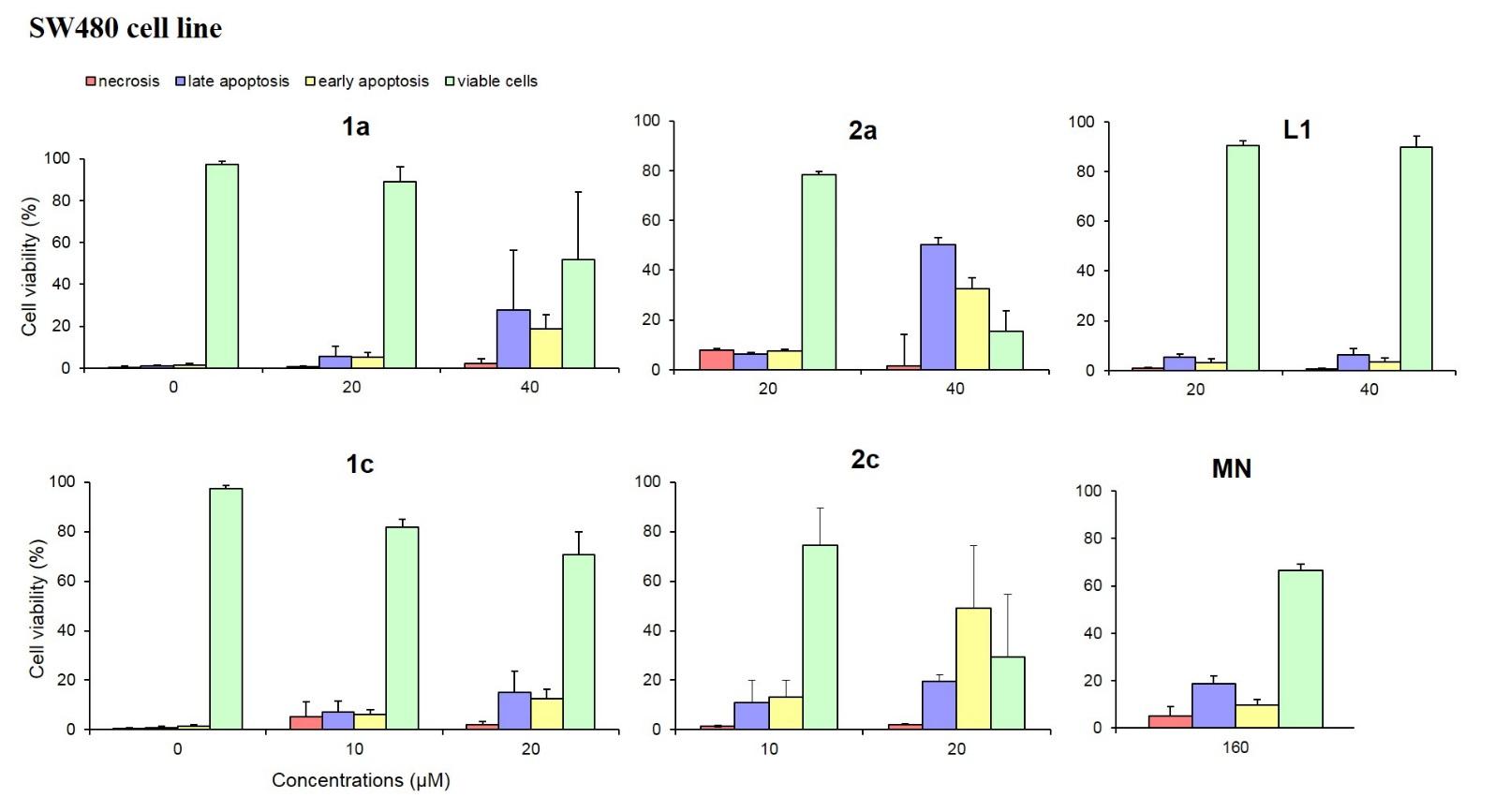


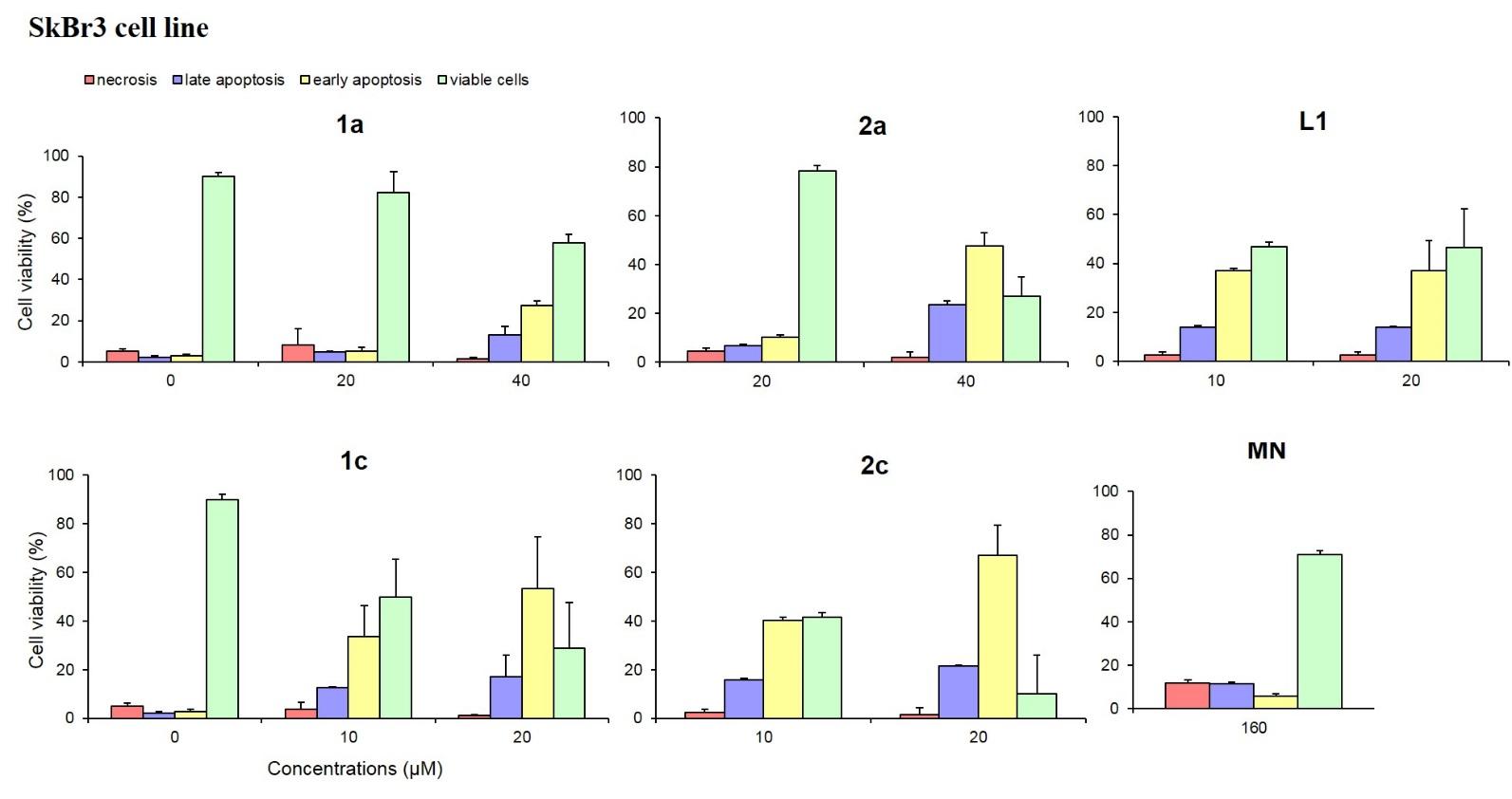


**Figure S3.** Apoptosis/necrosis induction in SW480, SK-BR-3, T47D and MDA cells after 48 h of exposure to the studied compounds, ligand and merbarone (MN); measured by flow cytometry using the annexin V-FITC/propidium iodide double staining (viable cells shown in green, early apoptosis – in yellow, late apoptosis – in violet and necrosis – in pink).


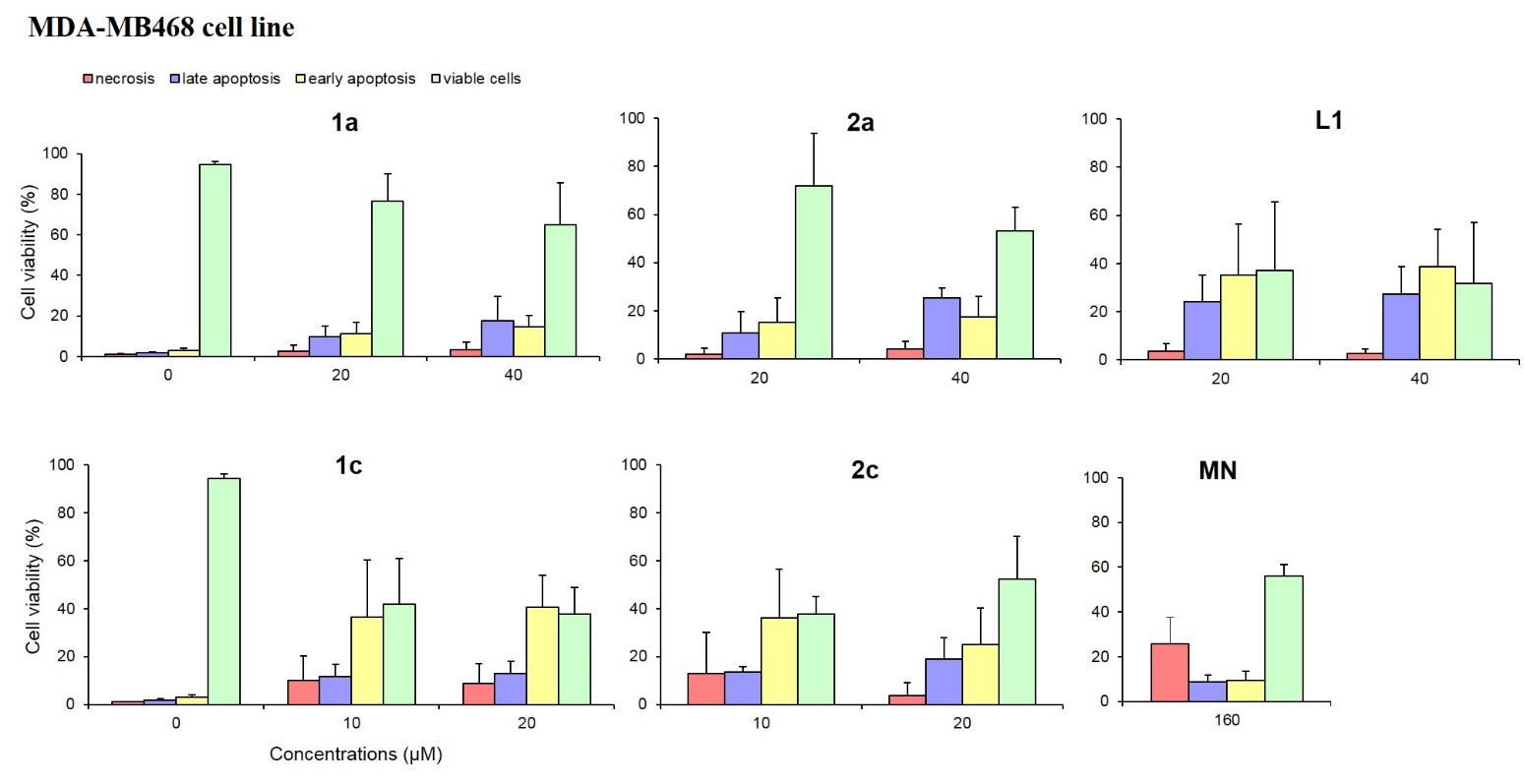


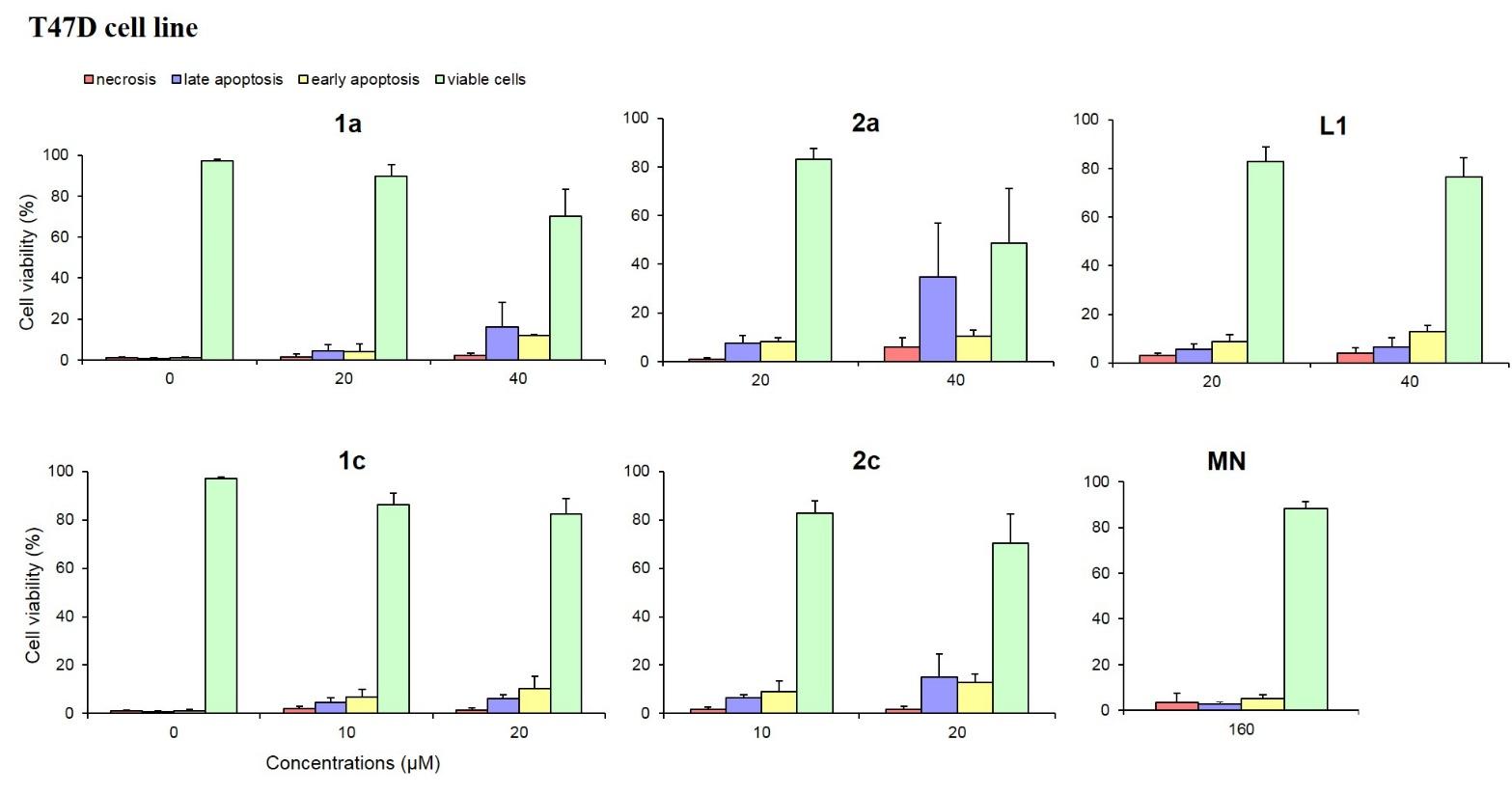


**Figure S3** (continued)
